# Functional Role of the microRNA-200 Family in Breast Morphogenesis and Neoplasia

**DOI:** 10.3390/genes5030804

**Published:** 2014-09-11

**Authors:** Bylgja Hilmarsdottir, Eirikur Briem, Jon Thor Bergthorsson, Magnus Karl Magnusson, Thorarinn Gudjonsson

**Affiliations:** 1 Stem Cell Research Unit, Department of Medical Faculty, Biomedical Center, University of Iceland, Vatnsmyrarvegi 16, 101 Reykjavík, Iceland; E-Mails: byh1@hi.is (B.H.); eib13@hi.is (E.B.); jon.bergthorsson@gmail.com (J.T.B.); magnuskm@hi.is (M.K.M.); 2 Department of Laboratory Hematology, Landspitali University Hospital, Hringbraut, 101 Reykjavík, Iceland; 3 Department of Pharmacology and Toxicology, Medical Faculty, University of Iceland, Hofsvallagotu 53, 107 Reykjavík, Iceland

**Keywords:** miRNAs, miR-200 family, breast, epithelium, branching morphogenesis, EMT, MET, breast cancer

## Abstract

Branching epithelial morphogenesis is closely linked to epithelial-to-mesenchymal transition (EMT), a process important in normal development and cancer progression. The miR-200 family regulates epithelial morphogenesis and EMT through a negative feedback loop with the ZEB1 and ZEB2 transcription factors. miR-200 inhibits expression of ZEB1/2 mRNA, which in turn can down-regulate the miR-200 family that further results in down-regulation of E-cadherin and induction of a mesenchymal phenotype. Recent studies show that the expression of miR-200 genes is high during late pregnancy and lactation, thereby indicating that these miRs are important for breast epithelial morphogenesis and differentiation. miR-200 genes have been studied intensively in relation to breast cancer progression and metastasis, where it has been shown that miR-200 members are down-regulated in basal-like breast cancer where the EMT phenotype is prominent. There is growing evidence that the miR-200 family is up-regulated in distal breast metastasis indicating that these miRs are important for colonization of metastatic breast cancer cells through induction of mesenchymal to epithelial transition. The dual role of miR-200 in primary and metastatic breast cancer is of interest for future therapeutic interventions, making it important to understand its role and interacting partners in more detail.

## 1. Introduction

Branching morphogenesis is a conservative process seen in several organs such as lung, prostate, kidney, salivary glands and breast. The unique role of branching morphogenesis is to increase surfaces of the epithelium/endothelium so it can deliver specific functions such as gas exchange and transport (lung, vasculature), urine formation/filtration (kidneys), and secretion from exocrine glands (prostate, salivary glands and breast). Developmental events underlying branching morphogenesis in epithelial tissues are closely related to pathways important for cancer progression and metastasis such as epithelial-to-mesenchymal transition (EMT) and its reverse process mesenchymal-to-epithelial transition (MET) [1,2,3,4]. Branching morphogenesis and EMT/MET are regulated by extrinsic and intrinsic factors. The mesenchyme and its cellular constituents are the dominant extrinsic factors regulating epithelial branching morphogenesis through secretion of soluble factors [1,5,6]. The extrinsic signals are received by their cognate receptors that convey the message to the appropriate intracellular pathways that finally activate or suppress their target transcription factors or other regulatory elements that switch on or off a particular phenotype. In recent years, we have seen significant progress in understanding the molecular regulation of cellular remodeling during organogenesis and tissue maintenance. Furthermore, due to the link between pathways regulating branching morphogenesis and cancer, there are intensive studies on the molecular regulation of these pathways [2,7]. miRs are increasingly seen as major intrinsic regulators of gene expression, and they have greatly extended our understanding of how differentiation and morphogenesis is regulated in the mammary gland and other organs [8,9]. miRs are single-stranded, 22 nucleotide-long molecules, which were first described in *Caenorhabditis elegans* [10]. To date, the number of annotated human miR loci is 1,881 for the human genome (miRBase v21) [11]. miRs are derived from primary miRs (pri-miRs), which are cleaved by the ribonuclease III (RNase III) enzyme Drosha into shorter hairpin structures called precursor-miRs (pre-miRs) that are ~60 nucleotides long [12]. Pre-miRs have a short stem and a two-nucleotide 3' overhang that is recognized by the nuclear transport receptor exportin 5 (EXP5), which exports them from the nucleus to the cytoplasm [13]. In the cytoplasm, another RNase III enzyme Dicer further processes the pre-miR into a ~22 nucleotide miR-miR* duplex [14]. The double-stranded RNA duplex is loaded into an Argonaute (AGO) protein and further processed, causing the miR* to be expelled, which results in a mature RNA-induced silencing complex (RISC), which can base pair to a target mRNA and induce its silencing [15]. A 6–8 nucleotide seed sequence at the 5' end of the miR is an important determinant for AGO binding to its target mRNA. Any region of an mRNA can have seed matches, but the matches in the 3'untranslated region (3'UTR) are more likely to decrease expression of mRNAs [16]. miRs play an important role in cellular processes by controlling essential steps such as proliferation, apoptosis, differentiation and morphogenesis [17]. Accumulating data show that miRs are also important regulators of stem cells and cell-fate decisions [18,19,20]. Furthermore, aberrant expression of a number of miRs is seen in various pathogenic processes including cancer [21].

miRs are now increasingly recognized as master regulators of protein expression through their ability to bind and degrade mRNA of various critical regulatory and signaling proteins, such as transcription factors involved in gene regulation.

**Figure 1:**
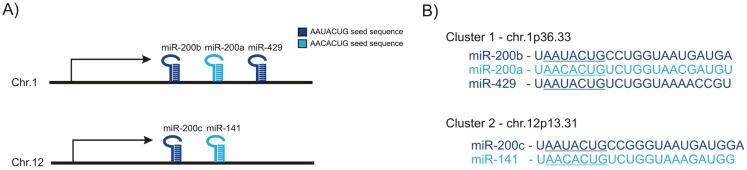
Genomic location and base sequence of the miR-200 family. (**A**) The miR-200 family is located on two distinct clusters on chromosome 1 and 12; (**B**) Sequences of the miR-200 family. The miR-200 family is divided in two functional groups by their seed sequence: miR-200a/141 and miR-200b/c/429.

The miR-200 family members have been demonstrated as being involved in regulation of many critical biological processes such as EMT/MET, stem cell and cancer stem cell regulation in addition to cell proliferation and drug resistance [22,23,24,25,26]. The miR-200 family is one of the best-known miR families in mammals [22,27]. It is located at two different loci at chromosomes 1 (miR-200b, miR-200a, and miR-429) and 12 (miR-200c and miR-141) (Figure 1A). All miR-200 family members contain a similar seed sequence with only one base difference between the two groups. miR-200c, miR-200b and miR-429 share the same seed sequence AAUACUG, whereas miR-200a and miR-141 contain AA**C**ACUG (Figure 1B). The seed sequence is the determining base sequence that decides which mRNA is targeted and degraded. miR-200b, miR200c, miR-429 (miR-200bc/429) and miR-200a and miR-141 (miR-200a/141) are functionally classified together based on their seed sequence. The miR-200 family is increasingly being recognized as an important regulator of epithelial integrity in the breast gland, and loss of expression has been linked to EMT and cancer invasion. It has also been linked to metastasis and its expression is believed to facilitate colonization of metastatic breast cancer cells at distant sites such as the lung through induction of MET. In this review, we will discuss the functional role of miR-200 family and in EMT and branching morphogenesis in the breast gland. We will also discuss the role of miR-200 in cancer invasion and colonization of metastatic breast cancer cells to distant organs.

## 2. miR-200 Family in Epithelial-to-Mesenchymal Transition

Cellular plasticity is crucial during embryogenesis, where tissue remodeling and cellular differentiation shape the architecture of the human body. The formation of the mesoderm from ectoderm during gastrulation is the first EMT event in development. EMT is also seen during neural crest formation and heart morphogenesis, as well as in wound healing [28]. In recent years there has been an increased focus on the role of EMT in cancer progression, in particular in the invasive and metastatic processes and in various fibrotic diseases [4,28,29].

In EMT induction, different pathways ultimately control transcriptional regulatory factors such as SNAIL1, SNAIL2, TWIST, ZEB1 and ZEB2, leading to increased expression of mesenchymal markers and decreased expression of epithelial markers [30]. The down-regulation of the epithelial cell-cell adhesion glycoprotein E-cadherin is one of the hallmarks of EMT [31]. In contrast, cells show increased expression of mesenchymal markers such as N-cadherin, vimentin, alpha smooth muscle actin and fibronectin [30].

The role of miR-200 in EMT has been studied intensively in recent years. miR-200 members are regulated by both transcription factors and by epigenetic regulation. Studies have identified a number of transcription factors that positively or negatively regulate its expression (reviewed in [22]) with ZEB1 and ZEB2 being the best known factors [25,27,32,33]. These transcription factors are responsible for down-regulation of miR-200 members resulting in the loss of E-cadherin expression and the subsequent EMT phenotype. Both ZEB1 and ZEB2 repress the expression of miR-200 family members through binding to regulatory Z- and E-boxes at their promoter area. ZEB1 has been shown to be able to repress the expression of miR-200c-141 and both ZEB1 and ZEB2 can repress the expression of the miR-200ba-429 cluster [34,35]. Interestingly, miR-200 members can also target ZEB1 and ZEB2 mRNA indicating a mutual feedback mechanism that may be important during branching morphogenesis where cells need to acquire a partial EMT until reaching the correct architecture through MET.

Epigenetic factors play an important role in regulating cell type specific gene expression, including miRs. Epigenetic mechanisms such as DNA methylation and histone modifications have been associated with regulation of the miR-200 family. A study comparing global miR expression and epigenetic states of human mammary fibroblasts and epithelial cells showed that fibroblasts repress the miR-200c/141 locus by both DNA methylation and dimethylation of histone 3 at lysine 9 (H3K9me2) and the miR-200ba/429 locus by promoter trimethylation of histone H3 at lysine 27 (H3K27me3). In contrast, the human mammary epithelial cells had their promoter regions occupied by trimethylation of histone H3 at lysine 4 (H3K4me3) and acetylation of histone H3 at multiple lysine residues (H3Ac), which are both marks of active transcription, while the fibroblasts were devoid of these marks [36].

Both miR-200 clusters are associated with local CpG enrichments. Wiklund *et al.* showed in 2011 that miR-200 were silenced with promoter hypermethylation and repressive chromatin marks in muscle-invasive bladder tumors and undifferentiated bladder cell lines [37]. DNA methylation at the miR-200c/141 promoter correlates highly with an EMT phenotype and invasive capacity of breast cancer cell lines [38]. Promoter hypermethylation at miR-200ba/429 and miR-200c/141 correlate with a mesenchymal phenotype, and overexpression of either of these clusters was shown to restore the epithelial properties of cells [39]. In human colon, the mesenchymal cells are methylated at both miR-200 loci, whereas the epithelial mucosa is not [39]. In a recent study, a model of cell transition between cancer stem-cell and non-stem-cell-like phenotype where gain of stem cell characteristics is followed by loss of miR-200 expression, epigenetic control of the miR-200 family was shown to be significantly altered in the transition, both with DNA methylation and histone modifications [40]. The miR-200ba/429 cluster was primarily silenced with histone modifications, whereas DNA methylation repressed miR-200c/141 expression. It is becoming clear that epigenetic control of miR expression, including the miR-200 family members, is an important tool for the cells to acquire correct fate decision.

In summary, transcription factors together with epigenetic modulation demonstrate the complex network of factors controlling the miR-200 family. Furthermore, the interactions of miR-200 with their targets show how complex the reciprocal communication between miR-200 and their regulators and targets are.

## 3. miR-200 Family in Branching Morphogenesis in the Mammary Gland

Branching morphogenesis is a highly conserved developmental process that occurs in many organs through similar signaling pathways and transcriptional regulation [41]. EMT has been linked to branching morphogenesis as it is widely accepted that during branching morphogenesis the epithelial cells need to invade the underlying matrix. The term epithelial plasticity (sometimes referred to as partial EMT (p-EMT)) has, however, more often been used to describe branching epithelial morphogenesis [42]. During p-EMT the invading cells acquire partial mesenchymal traits while retaining many critical epithelial properties. Another term used to describe these processes is collective migration, where tipping epithelial cells in the branching structures invade the underlying matrix in a collective pattern, indicating that the migrating cells adhere together through the migrating process [43]. There is a growing understanding that the invading cells need to acquire certain properties of mesenchymal cells to be able to invade the underlying matrix [44,45,46].

The mammary gland is unique in that most of its development occurs postnatally, and the epithelial remodeling can be followed from the onset of puberty, during each menstruation cycle and in more dramatic ways through each period of pregnancy and lactation [47]. Although the role of the miR-200 family in cancer has been studied extensively (see below), its role in branching morphogenesis has been studied to a lesser extent. Due to the importance of the miR-200 family in EMT and the fact that epithelial plasticity and p-EMT are important mechanisms in branching morphogenesis, it is likely that miRs-200 are central players in branching morphogenesis.

There are several studies that have focused directly or indirectly on the role of miRs-200 in mammary gland morphogenesis. Analyses of miR-200 expression in tissue from reduction mammoplasty show that the expression of miR-200 members are predominantly in the luminal epithelial compartment and to a much lesser extent in myoepithelial cells [48]. Although myoepithelial cells share the same origin as the luminal epithelial cells [49,50,51], they have acquired some mesenchymal traits such as expression of vimentin and α-smooth muscle actin that may explain the reduced expression of miR-200 in these cells. The function of the miR-200 family in human luminal breast epithelial cells is, however, currently unknown. Shimono *et al.* have shown that all miR-200 members are down-regulated in human and mouse mammary epithelial stem/progenitor cells and up-regulated in differentiated epithelial cells [26]. In this study the miR-200c was shown to regulate mammary epithelial differentiation through repression of the stem cell self-renewal gene BMI1. In addition, miR-200c overexpression in mouse mammary epithelial stem cells suppressed or impaired normal mammary ductal outgrowth *in vivo*. Recently, Song *et al.* demonstrated that miR-22 regulates side branching of mammary duct *in vivo* in transgenic mice through silencing of TET (Ten eleven translocation) family of methylcytosine dioxygenases, resulting in demethylation of the miR-200 promoter. Interestingly, the cooperation between miR-22 and the miR-200 family resulted in EMT, an elevated pool of stem cells and later increased tumorigenesis [52]. However, it is not known if inhibition of TET can alter global methylation profiles of cells. In a screening study, Avril-Sassen *et al.* demonstrated that seven temporally co-expressed miRNA clusters were implicated in regulating murine mammary gland development [53]. Global reduction in miRNA expression during lactation and early involution was reported, except for one of the clusters which showed the highest expression during these developmental stages [53]. Interestingly, this cluster contains members of the miR-200 family. Another screening study where Le Guillou *et al.* focused on miRs involved in mammary reproductive cycles, indicated that the miR-200 family is highly expressed during lactation [54]. Additionally, members of the miR-200 family were shown to be expressed during ovine pregnancy with increased expression at the end of pregnancy and during lactation [55]. These studies suggest that members of the miR-200 family preserve epithelial integrity in the mammary gland during maximal differentiation that occurs during lactation.

The role of miR-200 family members in other branching organs has received little attention so far. Rebustini *et al.* [56] screened miRs expressed in the mouse developing submandibular gland (SMG) and found that miR-200c accumulates in the epithelial end buds. Using both loss- and gain-of-function, they demonstrated that miR-200c reduces epithelial proliferation during SMG morphogenesis. Furthermore, they showed that miR-200c targets the very low density lipoprotein receptor (Vldlr) and its ligand reelin, which regulates FGFR-dependent epithelial proliferation [56]. In mouse kidneys, the miR-200 family has been implicated in renal tubular maturation, where miR-200-deficient collecting duct cells had 65% less tubulogenesis in a 3D collagen assay [57].

Although only few studies have shown any relevance for miR-200 family in branching morphogenesis in the mammary epithelium, they do suggest that miR-200 play a major regulatory role in maintaining epithelial integrity in the mammary gland. Most of these studies, however, have been conducted in the mouse mammary gland. There are substantial differences between mice and humans regarding mammary epithelial branching and the interaction between mammary epithelial and stromal cells. A better understanding of the role of these miRs within the human breast gland is needed and 3D culture models of human breast epithelial cells can substantially contribute to this field. We have generated a human breast epithelial cell line, D492, with stem cell properties. D492 generates luminal and myoepithelial cells and in 3D culture it is able to form elaborate bilayered branching structures with both myoepithelial and luminal cells reminiscent of the terminal duct alveolar unit in the human female breast gland [51,58]. When D492 is cultured in endothelial-rich stroma (matrigel), increased epithelial branching is observed indicating that endothelial cells are important stromal-derived inducers of branching morphogenesis [59]. Interestingly, we have recently observed that endothelial cells induce branching morphogenesis of epithelial cells from other organs such as lung and prostate [60,61]. Furthermore, we have also shown that endothelial cells induce epithelial-to-mesenchymal transition in D492 cells [59]. Current studies in our laboratory focus on understanding the role of miRs, including the miR-200 family in breast epithelial branching morphogenesis using the organtypic human branching morphogenesis model in 3D culture.

## 4. mir-200 Family and Breast Cancer

In recent years, miRs have increasingly been linked to functions that are either tumor promoting and/or tumor suppressing, and altered expression of miRs has been noted at various stages of cancer progression in different tissues [21,62,63,64]. Changes in the expression of miR-200 family members have been documented in various types of cancer, including cancer of the lung, ovary, stomach, endometrium and breast [65,66,67,68,69]. In many cases, these changes reflect hypermethylation of miR-200 family promoters [36,37,39]. The requirement for epithelial integrity in the human breast and the link between metastatic cancer and the EMT process has led to many clinically oriented studies focusing on the miR-200 family.

**Figure 2:**
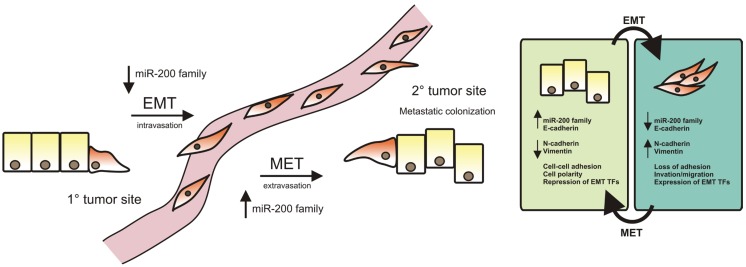
miR-200 family expression in EMT and MET. In EMT, expression of EMT transcription factors (TFs) leads to increased expression of mesenchymal markers and decreased expression of epithelial markers, followed by down-regulation of the cell adhesion and loss of polarity. With increased migration/invasion these mesenchymal-like cancer cells enter the blood or lymphatic vessels and travel to a secondary site, where they go through MET to form metastatic colonization. During the initial phase of local tumor growth, the miR-200 members maintain epithelial integrity and can thus suppress or reverse cancer progression. In contrast, once the disease has spread to distant organs, miR-200 may potentially accelerate tumor progression by facilitating re-adaptation of the epithelial phenotype and colonization.

In breast cancer, previous cluster analysis of gene expression data has identified several different subtypes including luminal (A and B), ErbB2 over-expressing and basal-like breast cancer [70,71]. The luminal types have a gene expression signature that infers luminal epithelial origin, and they are positive for estrogen and progesterone receptors. The expression profile of ErbB2 tumors is largely shaped by over-expression of the ErbB2 tyrosine kinase receptor that commonly occurs due to gene amplification. Basal-like tumors express genes that determine basal and myoepithelial phenotype. Further categorization of the basal phenotype according to marker expression, invasive behavior and mesenchymal morphology has yielded additional subtypes including the claudin-low subtype [72]. The basal-like breast tumors are richer in mesenchymal and EMT features and are associated with metastasis and aggressive clinical behavior [73] (Figure 2). In concurrence with its relevance to EMT, the luminal types express higher level of the miR-200 family than ErbB2 overexpressing and basal-like breast cancer [74]. Accordingly, levels of miR-200 family targets such as SNAI1, SNAI2, and ZEB2 are higher in these tumors. However, promoter methylation only explains suppression of the miR-200/141 locus in metaplastic cancer [74]. Of the individual miRs of the miR-200 family, expression levels of miR-200c seem to be the most strongly associated with the histopathology and clinical course of breast cancer, whereby miR-200c levels are down-regulated early in the formation of ductal carcinoma *in situ* (DCIS) and almost absent in TN cancers [75,76].

Although the miR-200 family is increasingly recognized as a family of tumor suppressors there are indications that its expression can improve survival of metastatic potential in cancer [77]. Korpal *et al.* recently demonstrated that the miR-200 family was important in distal metastasis of breast cancer cells by facilitating epithelial colonization in lung through induction of mesenchymal to epithelial transition (MET) [78] (Figure 2). In this study, they demonstrated that miR-200 induced MET, including reestablishment of E-cadherin expression, and was independent of ZEB1 expression but relied on the ER–Golgi protein trafficking protein Sec23a to rescue the epithelial phenotype. This suggests a dual role for miR-200 family members in breast cancer progression. During the initial phase of local tumor growth, the miR-200 members suppress or reverse cancer progression [79]. In contrast, once the disease has spread to distant organs, miR-200 may potentially accelerate tumor progression by facilitating re-adaptation of the epithelial phenotype and colonization. Dykxhoorn *et al.* worked with four isogenic mouse breast cancer cell lines that differ in their ability to metastasize [77]. The only cell line that expressed the miR-200 family was the 4T1 cell line, which was also the only cell line able to form lung and liver metastases. When 4T1 and the non-metastatic 4TO7 cells where implanted into a mammary fat pad, 4T1 cells formed tumors more rapidly than 4TO7 cells. To study the role of miR-200c/141 in metastasis, Dykxhoorn *et al.* transduced 4TO7 with a vector stably expressing the miR-200c/141 cluster. The 4TO7 cells stably expressing miR-200c/141 formed metastases at the same rate as the 4T1 cells, showing that miR-200c/141 expression was sufficient to make these cells metastatic [77].

Using an *in vivo* cycling strategy, Banyard *et al.* selected metastatic cancer cells from the lymph nodes (LN) of mice bearing orthotopic DU145 human prostate tumors [80]. Repeated rounds of metastatic selection (LN1–LN4) progressively increased the epithelial phenotype, resulting in a new model of tumor cell mesenchymal-epithelial transition (MET). This was accompanied by increased expression of the miR-200 family. Furthermore, they showed that miR-200c inhibition caused EMT, with increased expression of both ZEB1 and ZEB2 and E-cadherin down-regulation [80].

Taken together, these data imply that complete loss of miR-200 family members, e.g., by genomic deletions as observed for classical tumor suppressor genes, may actually predict a favorable clinical outcome. The genomic location of the miR-200 family genes is on chromosome 1p36 (miR-200ba/429), a locus associated with a moderate frequency of genomic deletions, and chromosome 12p13 (miR-200c/141), associated with equally moderate frequency of DNA copy number gains and deletions in human breast cancer [81].

In line with the above discussion, induction of miR-200 family expression as a strategy for therapy needs to be directed at early phases of the disease. Wang *et al.* showed recently that the drug tetraindole SK228 induces miR-200c expression in breast cancer cells followed by repression of ZEB1 and ZEB2 and re-expression E-cadherin [82]. The cells reestablish the epithelial phenotype, and *in vivo* studies indicate reduced breast cancer growth making this drug a potential agent for future cancer therapy. Direct administration of miR-200 family members for therapeutic purposes can at least be considered since miR-based drugs already have entered clinical trials [83]. Recently, the miR-200 family was shown to therapeutically inhibit angiogenesis in experimental models [84]. In this study Peco *et al.* showed that miR-200 family members directly target the pro-angiogenic cytokines IL-8 and CXCL1. A systemic miR-200 delivery resulted in marked reductions in metastasis had anti-angiogenic effect in several different cancer mouse models. Importantly, Peco *et al*. [84] found that miR-200 expression was associated differently to patient survival depending on cancer type, suggesting that miR-200 effects on prognosis is context dependent. Apart from protection against pathogenic EMT, there may be other advantages of miR-200 family member up-regulation for clinical use, e.g., miR-200c seems to sensitize TN breast cancers to cell death [85]. In addition, miR-200b may also have potential as a therapeutic agent since it has been shown to inhibit metastatic growth in TN breast cancer through inhibition of protein kinase Cα (PKCα) [86]. Recently, PKCα inhibition was shown to specifically target cancer stem cells with little effect on the non-cancer stem cells in basal-like breast cancer cell lines, which could prove to be an effective approach in treating aggressive cancers [87]. Alternative to the administration of the miR-200 family members in therapy, it could be feasible to inhibit their downstream pathways using enzyme inhibitors.

These data along with the above discussion suggest a dual role for miR-200 family members in breast cancer. In the initial phase of breast cancer, while the tumor is still local, the miR-200 members could be potential therapeutic agents by inhibiting or reversing the malignant progression. In contrast, if the tumor cells have already metastasized to distinct organs, the miR-200 members could possibly accelerate the tumor progression by helping cells to colonize in distant environments/organs. Further studies on the multiple functions of the miR-200 family are warranted before they can be claimed relevant as potential agents or targets of cancer therapy.

## 5. Future Perspective

miRs are major regulators of cell fate through their ability to inhibit gene expression. It is becoming clear that the miR-200 family is associated with epithelial tissue, and their role is to maintain the epithelial integrity in normal tissue through stimulation of cell-cell interactions, including induction of E-cadherin expression. In the breast gland, miR-200 family members are predominantly expressed in the luminal epithelial compartment. The fact that miR-200 expression goes down during EMT and up again during MET, make the miR-200 family an interesting candidate as a regulator of cancer progression and a possible therapeutic target. In terms of cancer progression, it is important to look at the expression in the temporal context, where expression in primary tumors may be beneficial in order to retain epithelial integrity, while expression in distal metastasis may facilitate colonization and tumor growth.

Studying the functional role of miRs in normal tissue morphogenesis, including branching morphogenesis and epithelial integrity in distinct organs including the breast gland, have the opportunity to shed light on how miRs contribute to normal development and disease progression. Further studies of the miR-200 family in the epithelial tissues are warranted due to their potential to retain and restore the epithelial integrity. It will be interesting to see how miR-200 family members regulate epithelial stem and progenitor cells. Are epithelial stem cells lacking miR-200 expression? What happens when miR-200 family members are introduced into breast epithelial cells with stem cell properties? Does this drive cells into luminal epithelial differentiation and/or block cell proliferation?

Cellular localization of miRs can be visualized using *in situ* hybridization. This is, however a difficult task in most cases because of the short sequence of each miR and the difficulties of getting the probes to bind in a specific manner. Compared to protein expression, which can be easily identified by specific antibodies, an ongoing challenge is to improve techniques to visualize expression patterns of distinct miRs in tissues and identify subcellular expression patterns. Recently Renwick *et al.* made important advances in this area in a study where they enhanced miRs detection in cancer samples by increasing linker lengths and signal amplification [88].

There is a gap in our current knowledge of the role of the miR-200 family in branching morphogenesis in the human breast, and there is a need for functional models to study its role in developmental processes in branching organs such as the breast. In that regard, organtypic 3D cell culture models could be of help. Even though the miR-200 family is recognized as a master regulator of epithelial integrity in cells, we also propose a need for expressional and functional analysis of different miR-200 members in distinct cell populations of the breast gland.

The ultimate goal of clinical cancer research is to reverse the malignant phenotype in tumors preventing the metastatic process. Although intrinsic factors such as the miR-200 family are powerful effectors of epithelial integrity, the extrinsic factors are also important inducers of epithelial fates. Extrinsic variables like growth factors or the extracellular matrix are strong initiators of signal transduction that in turn result in activation of intracellular factors such as miRs. It is of great importance to identify the extrinsic factors that influence the transcription regulation of different miRs. There are a number of agents that have been shown to revert the malignant phenotype towards a more benign state. Weaver *et al.* showed in 3D culture that blocking overexpressed β1-integrin in a HMT3522-T4 cancer cell line could revert the phenotype to more polarized normal-like acini [89]. They later showed that this occurs through down-regulation of the MAPK kinase pathway and EGFR expression [90].

Truong *et al.* [91] found recently that the loss of β1-integrin represses primary breast tumor growth but stimulated metastasis to the lungs. Antibodies that blocked β1-integrin function switched the migratory behavior of human and mouse E-cadherin positive triple-negative breast cancer (TNBC) cells from collective to single cell movement. Interestingly, this switch activated the transforming growth factor-beta (TGF-β) signaling pathway that led to a shift in the balance between miR-200 and ZEB2, resulting in suppressed transcription of the gene encoding E-cadherin expression [91]. Many of these studies were performed before the biological importance of miRs was known. It is likely that miRs are interwoven with many of these processes and could help to explain the molecular pathways leading to either tumor progression and or reversion of the malignant phenotype.

## 6. Conclusions

It is becoming clear that the existence of miRs has revolutionized our understanding of various processes in cell and molecular biology. By increasing our understanding of the functional role of each miR, we are now better able to put signaling pathways and transcription regulation into broader context. miRs are stepping in as master regulators of many developmental processes, and our understanding of the complexity of their function is also rapidly expanding. The miR-200 family is being recognized as a guardian of epithelial integrity in normal tissue including the breast. Its role in preventing EMT has been solidly evidenced and, in normal breast and primary cancer, miR-200 has been considered a tumor suppressor. The recent data demonstrating that miR-200 is up-regulated in breast cancer metastasis needs to be taken into contextual evaluation before therapy for knock in or out miR-200 members is initiated.
